# Comparison of the In Vivo Biotransformation of Two Emerging Estrogenic Contaminants, BP2 and BPS, in Zebrafish Embryos and Adults

**DOI:** 10.3390/ijms18040704

**Published:** 2017-03-25

**Authors:** Vincent Le Fol, François Brion, Anne Hillenweck, Elisabeth Perdu, Sandrine Bruel, Selim Aït-Aïssa, Jean-Pierre Cravedi, Daniel Zalko

**Affiliations:** 1 Institut National de l’Environnement Industriel et des Risques (INERIS), Unité d’Écotoxicologie In Vitro et In Vivo, F-60550 Verneuil-en-Halatte, France; vincent.lefol@efs.sante.fr (V.L.F.); selim.ait-aissa@ineris.fr (S.A.-A.); 2 Toxalim (Research Centre in Food Toxicology), Université de Toulouse, INRA, ENVT, INP-Purpan, UPS, 31027 Toulouse, France; Anne.Hillenweck@inra.fr (A.H.); perduptoul@aol.com (E.P.); sandrine.bruel@inra.fr (S.B.); jean-pierre.cravedi@inra.fr (J.-P.C.)

**Keywords:** metabolism of xenobiotics, zebrafish, endocrine disruptors, benzophenone-2, bisphenol S

## Abstract

Zebrafish embryo assays are increasingly used in the toxicological assessment of endocrine disruptors. Among other advantages, these models are 3R-compliant and are fit for screening purposes. Biotransformation processes are well-recognized as a critical factor influencing toxic response, but major gaps of knowledge exist regarding the characterization of functional metabolic capacities expressed in zebrafish. Comparative metabolic studies between embryos and adults are even scarcer. Using ^3^H-labeled chemicals, we examined the fate of two estrogenic emerging contaminants, benzophenone-2 (BP2) and bisphenol S (BPS), in 4-day embryos and adult zebrafish. BPS and BP2 were exclusively metabolized through phase II pathways, with no major qualitative difference between larvae and adults except the occurrence of a BP2-di-glucuronide in adults. Quantitatively, the biotransformation of both molecules was more extensive in adults. For BPS, glucuronidation was the predominant pathway in adults and larvae. For BP2, glucuronidation was the major pathway in larvae, but sulfation predominated in adults, with ca. 40% conversion of parent BP2 and an extensive release of several conjugates into water. Further larvae/adults quantitative differences were demonstrated for both molecules, with higher residue concentrations measured in larvae. The study contributes novel data regarding the metabolism of BPS and BP2 in a fish model and shows that phase II conjugation pathways are already functional in 4-dpf-old zebrafish. Comparative analysis of BP2 and BPS metabolic profiles in zebrafish larvae and adults further supports the use of zebrafish embryo as a relevant model in which toxicity and estrogenic activity can be assessed, while taking into account the absorption and fate of tested substances.

## 1. Introduction

The involvement of endocrine-disrupting chemicals (EDCs) in the onset of adverse developmental and reproductive health effects in human and wildlife has been extensively documented, underlining the necessity to characterize the risk related to these compounds [1,2]. In this context, mammalian and non-mammalian in vitro and in vivo species-specific bioassays have been developed and implemented for the screening and testing of the endocrine activity of chemical substances, with both research and regulatory perspectives [3].

Zebrafish (*Danio rerio*) is now recognized as a valuable biological model for research in a variety of biological disciplines, from basic developmental biology to applied (eco)-toxicology. Notably, zebrafish has been found to be useful in studies related to EDCs, with important research efforts conducted over the past few years. This led to significant advances in the understanding of the mode of action of EDCs on steroid receptor-regulated pathways, and of the development of both in vitro and in vivo biological models and assays used to assess the endocrine disrupting (ED) potency of chemicals [4]. Regarding EDCs which act through the Estrogen Receptor (ER) signaling pathways, new zebrafish-specific in vitro and in vivo reporter gene assays have recently been developed. These are, on the one hand, liver cell lines (ZFL) stably transfected with the zebrafish estrogen receptor subtypes (ZELH-zfERs cell lines) expressing luciferase under the control of the estrogen responsive elements (ERE) [5] and, on the other hand, the EASZY assay (detection of Endocrine Active Substance, acting through estrogen receptors, using transgenic *cyp19a1b*-GFP Zebrafish embryos), based on transgenic zebrafish embryos expressing the Green Fluorescent Protein (GFP) under the control of the ER-regulated brain aromatase *cyp19a1b* promoter [6]. The combined use of these in vitro and in vivo methods in a tiered-approach is quite promising for the assessment of the estrogenic activity of chemicals in fish, since they provide reliable and accurate information on the molecular mode of action and the effect of substances on the endocrine system.

Like other chemicals, EDCs may be activated or deactivated through biotransformation carried out by xenobiotic metabolizing enzymes (XME). The fate of xenobiotics within biological models is a key factor in toxicity assessment [7] and it is acknowledged that the nature and concentration of active compounds ultimately reaching cellular targets is largely determined by metabolic processes. Ideally, in vitro and in vivo models should mimic as closely as possible the metabolic fate of candidate EDCs in wildlife species and/or humans. We recently characterized the comparative fate of the candidate EDCs bisphenol S (BPS) and benzophenone-2 (BP2) in human and zebrafish cellular models [8,9,10]. Benzophenones are emerging environmental contaminants used as UV filters in sunscreens and in other products such as perfumes or food packaging, to which they are incorporated to prevent UV degradation. BP2 exhibits EDC properties in fish [5,11,12], in mice [13] and in human models [14]. BPS, a molecule increasingly used as a substitute for bisphenol A (BPA), was also shown to be an estrogenic compound in fish and human models [15,16,17]. The comparative metabolic study of BP2 and BPS in primary zebrafish hepatocyte cultures, and in ZFL and ZELH-zfERs cell lines, demonstrated that these cellular systems are metabolically competent [8]. Fish embryo assays may provide an alternative small scale analysis system with a complexity close to an adult organism [18,19]. However, our current knowledge about the biotransformation capacities expressed by zebrafish at different developmental stages remains very fragmented due to a lack of information about the fate of model chemicals, and such information could explain differences in the estrogenic activity measured for candidate EDCs. Whether adult fish can absorb and metabolize environmental contaminants is not a matter of debate [20,21]. The early developmental expression of the majority of cytochromes P450 (CYPs) including those coding for xenobiotic metabolizing enzymes (XME) has been demonstrated [22,23], and the spatiotemporal distribution, modulation and activity of major zebrafish CYPs was recently reviewed in detail by Saad and co-workers [24]. Demonstrating the functionality of xenobiotic biotransformation systems during the early development of zebrafish is even more of a more challenging task. Using an in vitro approach based on the use of sub-cellular microsomal fractions and of specific probe substrates, Verbueken et al. [25] concluded that the capacity of intrinsic biotransformation in zebrafish embryos appears to be lacking during a major part of organogenesis, at least concerning key phase I activities. Notwithstanding, recent in vivo studies demonstrated that biotransformation of some compounds can start very early, indicating that some phase I and phase II enzymes are already functional at early life-stages [26,27]. We previously demonstrated that the metabolic capacities expressed in zebrafish hepatic cellular models are close to that of metabolically competent hepatic human cell lines (HepaRG), at least for BPS and BP2 [8]. However, no evidence was provided (nor is currently available in the literature) to assess whether the biotransformation of model EDCs in zebrafish embryos accurately reflects adult fish metabolism, and the fate of these EDCs in other vertebrates, including human. Using ^3^H-labeled molecules, we investigated in the current study the comparative fate of BP2 and BPS in larvae versus adult zebrafish. Metabolic balance and biotransformation experiments were carried out in vivo, as well as the radio-HPLC (High Performance Liquid Chromatography) profiling and characterization of metabolites present in water as well as in zebrafish themselves.

## 2. Results

No mortality and no signs of animal suffering were observed during the BP2 and BPS exposure experiments. All water samples and extracts of larvae as well as adult zebrafish, were analyzed by radio-HPLC for metabolic profiling, and were analyzed again after specific enzymatic hydrolysis tests for metabolite identity confirmation (control of radioactive peaks deconjugation).

### 2.1. Radioactivity Metabolic Balance

#### 2.1.1. Exposure of Zebrafish Larvae to Bisphenol S (BPS) and Benzophenone-2 (BP2)

Total radioactivity recovery (exposure water + beaker rinses + larvae extracts) averaged 102.7% ± 1.8% (BPS) and 102.0% ± 2.4% (BP2) of the nominal amount of radioactivity initially added to incubation beakers. Most of the radioactivity was recovered in water samples (BPS: 100.6% ± 2.4%; BP2: 99.2% ± 3.7%), while larvae themselves contained 0.22% ± 0.06% (BPS) and 0.90% ± 0.07% (BP2) of the radioactivity at the end of the 72 h incubation period. On wet weight bases, these values corresponded to 5.6 ± 1.0 µg of BPS equivalents and to 22.9 ± 1.2 µg of BP2 equivalents per gram of larva, respectively. For water samples, the calculated concentrations were 251.8 ± 3.0 µg of BPS equivalents and 244.3 ± 4.5 µg of BP2 equivalents per liter of water, respectively. Thus, the apparent bioconcentration factors were 22.0 ± 3.8 for BPS and 93.3 ± 5.0 for BP2.

#### 2.1.2. Exposure of Adult Zebrafish to BPS and BP2

Radioactivity recovery (exposure water + tank rinses + adult extracts and extracts pellets) averaged 100.3% ± 0.2% (BPS) and 99.7% ± 0.4% (BP2) of the nominal amount of radioactivity added to incubation tanks. As observed in larvae experiments, most of the radioactivity was recovered in water samples (99.7% ± 0.03% for BPS, 99.1% ± 0.2% for BP2). At the end of the 72 h exposure period, radioactivity in fish extracts accounted for 0.29% ± 0.03% (BPS) and 0.89% ± 0.20% (BP2) of the nominal radioactivity. On wet weight bases, these values corresponded to 0.14 ± 0.01 µg of BPS equivalents per gram of adult extracts, and to 0.43 ± 0.1 µg of BP2 equivalents per gram of adult extracts. The calculated concentrations of residues in water were 249.6 ± 0.1 µg of BPS equivalents per liter of water and 243.9 ± 5.4 µg of BP2 equivalents per liter of water. Apparent bioconcentration factors were thus 0.6 ± 0.1 for BPS and 1.7 ± 0.4 for BP2.

#### 2.1.3. Metabolic Profiling in Zebrafish Adults and Larvae Exposed to BPS

**Figure 1:**
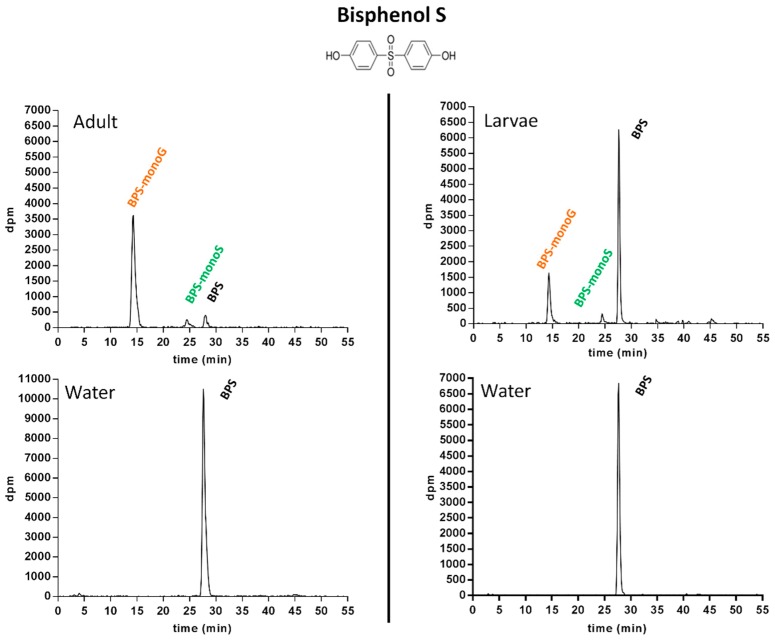
Typical radio-HPLC metabolic profiles obtained following the analysis of fish extracts and incubation water samples, after a 72 h incubation of 1 µM [3H-BPS] with adult or larvae zebrafish.

All water and extract samples were profiled, with the exception of the dichloromethane phases of adult extracts, which were found to contain a very minor part of the radioactivity put in incubations (BPS: <0.03%; BP2: 0.11%). Accordingly, for adult extracts, only the aqueous phase was further analyzed. Based on the radio-HPLC system developed for BPS in a previous work [8], the retention time (*R*_T_) of parent BPS was 27.7 min (Figure 1).

No degradation of BPS occurred in control incubations. In extracts from larvae as well as adult zebrafish, two conjugated metabolites of BPS were detected. The metabolite eluting at a *R*_T_ of 14.6 min was found to be fully hydrolyzed into BPS by β-glucuronidase, suggesting the occurrence of a BPS-glucuronide. The second metabolite, eluting at a *R*_T_ of 24.5 min, was deconjugated into BPS when incubated with sulfatase, suggesting the presence of a sulfate conjugate. No deconjugation occurred in control incubations, neither when the 14.6 min *R*_T_ metabolite was submitted to sulfatase hydrolysis, nor when the 24.5 min *R*_T_ metabolite was incubated with glucuronidase. This allowed the hypothesis of doubly conjugated (sulfate + glucuronide) metabolites to be ruled out. Finally, the structures of BPS mono-glucuronide (*R*_T_ 14.6 min; BPS-monoG) and of BPS mono-sulfate (*R*_T_ 24.5 min; BPS-monoS) were confirmed by co-elution assays with their respective authentic standards previously isolated from zebrafish hepatocyte incubations, and whose structure had been confirmed by liquid chromatography coupled to mass spectrometry (LC-MS), as detailed in Le Fol et al., (2015) [8]. In the water samples corresponding to adults as well as larvae assays, only the parent compound was detected (Figure 1, lower section). In extracts from larvae, 65.8% ± 5.3% of the radioactivity put in incubations was recovered as unchanged (parent) BPS at 72 h. For adults, the biotransformation of BPS was far more extensive, with only 12.1% ± 4.3% of the radioactivity recovered as parent compound in extracts by the end of the experiments (Figure 1).

#### 2.1.4. Metabolic Profiling in Zebrafish Adults and Larvae Exposed to BP2

**Figure 2:**
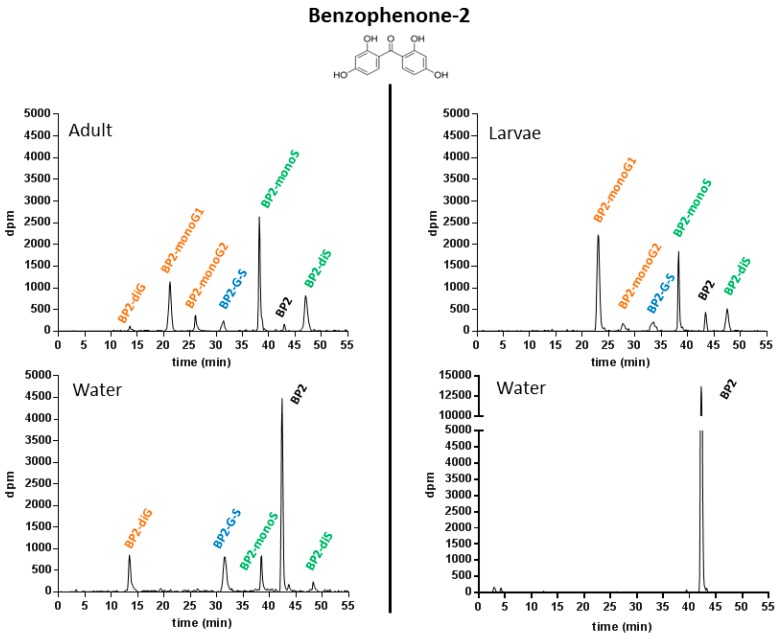
Typical radio-HPLC metabolic profiles obtained following the analysis of fish extracts and incubation water samples, after a 72 h incubation of 1 µM [3H-BP2] with adult or larvae zebrafish.

The HPLC system used was based on the original radio-HPLC system developed for BP2 by Le Fol et al., (2015) [8]. With this system, parent (unchanged) BP2 was eluted at *R*_T_ 42.5 min (Figure 2).

As for BPS, no degradation of BP2 was observed in radio-HPLC analyses of control incubations, with the exception of a very minor ^3^H exchange, observable on the radio-chromatograms of water samples (<1%). In adult extracts, three different glucuronide conjugates were identified. The metabolite eluted first (*R*_T_: 13.8 min) was a diglucuronide of BP2 (BP2-diG), based on specific enzymatic deconjugation assays, followed by radio-HPLC co-elution with the authentic standard, whose structure had previously been confirmed by LC-MS [8]. The two other metabolites, eluted at *R*_Ts_ of 22.1 and 27.3 min, respectively, were identified as two distinct mono-glucuronides (BP2-monoG1 and BP2-monoG2, respectively) based on complete deconjugation into BP2 when incubated with β-glucuronidase, and on their co-elution with their respective authentic standards, previously isolated and confirmed by LC-MS experiments [8]. The occurrence of two different mono-glucuronides is easily explained by the presence of two hydroxyl groups in the para and ortho position on each cycle of the BP2 molecule. The metabolite eluted at 38.1 min was characterized as a BP2 monosulfate (BP2-monoS), based on successful sulfatase hydrolysis. Its structure was confirmed by co-elution with the previously isolated standard metabolite characterized by LC-MS. The occurrence of BP2 di-sulfate (*R*_T_: 22.1 min) was suggested by co-elution with the authentic standard synthesized in parallel in incubations of BP2 with guinea pig cytosols, but the amounts of metabolite produced by fish did not allow a direct MS confirmation. Finally, the metabolite eluted at 32.8 min was tentatively identified as another double conjugate of BP2 (glucuronic acid + sulfate; BP2-G-S), based on specific enzymatic hydrolyses (deconjugation into BP2-G in incubations carried out with sulfatase, and into BPS-S in incubations carried out with glucuronidase, respectively). In larvae extracts, the same metabolites, with the exception of BP2-diG, were found to be formed (Figure 2, upper right). For BP2, all the metabolites detected and identified in adult extracts were also found to be present in water samples, with the exception of the two BP2 mono-glucuronides. The excretion of BP2 metabolites into water demonstrated an extensive metabolism of BP2 in adults compared to larvae. For the latter, water samples contained only unchanged BP2 (Figure 2, lower right). This was also a major difference compared to BPS, for which no excretion of metabolites into water occurred, even in adults.

#### 2.1.5. Pattern of BPS and BP2 Biotransformation in Zebrafish Adults and Larvae

**Figure 3:**
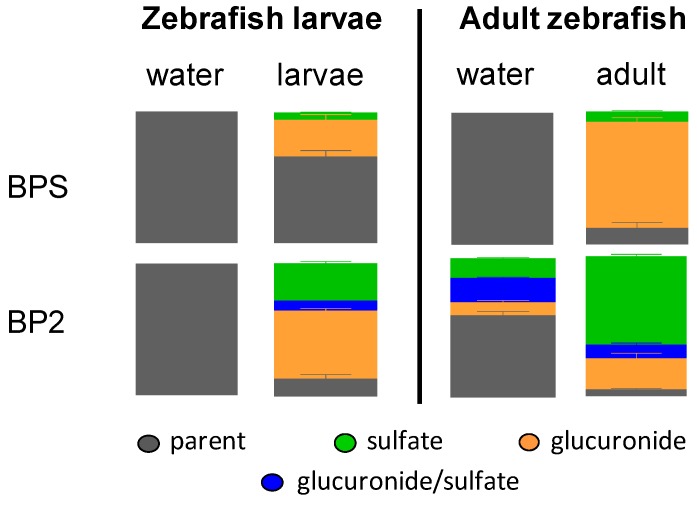
Metabolic balance of ^3^H-BPS and ^3^H-BP2 in larvae and adult zebrafish: respective proportions of parent molecules and their metabolites in water and animals samples at 72 h.

Only parent BPS was found to be present in water samples from experiments carried out with either adults or larvae (Figure 3). Conversely, BPS metabolites accounted for one-third of the radioactivity remaining inside larvae at the end of the study, and more than five-sixths of it in adult animals. In both larvae and adults, BPS-monoG was the major metabolite (28.1% ± 4.8% and 78.5% ± 3.3%, respectively), while BPS-monoS was detected in lower amounts in larvae (5.7% ± 1.0%) than in adults (7.7% ± 0.7%). Results for BP2 were clearly different. In adult zebrafish incubations, but also in larvae incubations, very low amounts of unchanged BP2 were found to be present in the aqueous phase of extracts (12.7% ± 3.7% and 4.5% ± 0.9%, respectively, Figure 3). BP2 metabolites produced in larvae were mainly glucuronide conjugates (51.3% ± 2.2%). Conversely, sulfate conjugates were found to be predominant in adult extracts (63.0% ± 1.6%). However, both conjugation pathways were active in larvae (28.4% ± 1.9% of BP2-sulfate and 7.4% ± 0.4% of BP2-glucurono-sulfate) as well as in adults (22.3% ± 3.7% of BP2-glucuronide and 9.7% ± 1.4% of BP2-glucurono-sulfate, Figure 3). For larvae, only unchanged BP2 could be detected in water. For adults, although water samples contained 56.2% ± 3.4% of parent BP2 by the end of the experiment, three metabolites, namely BP2-glucuronide (9.2% ± 1.5%), BP2-sulfate (13.6% ± 0.8%) and BP2-glucurono-sulfate (16.6% ± 0.9%) were identified as well (Figure 2 and Figure 3). As for both models (larvae, adults), metabolic balance results had shown that most of the radioactivity (>99%) was located in water samples; these results in adults demonstrate that mature zebrafish extensively metabolized BP2.

## 3. Discussion

Zebrafish embryos represent a very promising model in the context of ecotoxicology as well as toxicology studies, with an increasing span of utilizations ranging from the prediction of fish acute toxicity to the identification of endocrine disrupting chemicals. This model is 3-Rs compliant (up to 5 days post fecundation) and offers screening possibilities. Notwithstanding these advantages, major knowledge gaps remain regarding the actual metabolic capacities expressed by zebrafish embryos and the comparison of these capacities with the situation in adults, thus limiting their optimal use in the context of the assessment of chemicals toxicity.

In the present study, we investigated the metabolism of BP2 and BPS in 4-dpf-old zebrafish and in adult zebrafish, using labeled molecules and radio-HPLC. BP2 and BPS share many chemical features, including the presence of two phenol rings, close pKa values (6.98 for BP2; 8.2 for BPS) and molecular weights (246.22 for BP2; 250.27 for BPS). Nevertheless, our study underlined important differences in the fate of these xeno-estrogens, regarding metabolic profiles as well as bio-concentration factors. The use of radio-labeled compounds provided unequivocal evidence for the absorption of both BP2 and BPS in zebrafish larvae as well as adults. Notably, the capacity of larvae at early stages of development (96 hpf) to uptake but also to biotransform these EDCs was demonstrated. For BPS, metabolic balance studies showed an uptake of 0.22% (larvae) and 0.29% (adults) of the radioactivity put in water, which is consistent with previous findings about bisphenol A (0.20% at 24 h) when using zebrafish larvae with a similar study design [28]. For BP2, global uptake of radioactivity was higher in larvae (0.90%) than in adults (0.57%). However, taking into account the respective biomasses, actual concentrations in fish were always markedly higher in larvae: BPS concentration in fish bodies was about 40-fold higher in larvae (5.6 µg/g) than in adults (0.14 µg/g), and BP2 concentration was above 50-fold higher in larvae (22.9 µg/g) than in adults (0.43 µg/g). These results clearly emphasize the tight interaction between fish and their environment, as regards the exposure to EDCs. Exposure can occur soon after hatching, even at non-feeding stages of development. While gills constitute an important pathway of exchange and of detoxification in adults, they are not yet functional in 96 hpf (h post fecundation) larvae. Zebrafish larvae possess a hollow intestine tube with an open mouth only by 74–76 hpf [29], but it seems that no chemical uptake from the digestive tract occurs before 120 hpf [19]. At theses stages of development, a passive diffusion from media into larvae represents the main exposure pathway [30,31]. This was likely the process involved in our study, for zebrafish larvae.

BPS and BP2 were exclusively metabolized through phase II pathways. Many among the chemicals produced in large volume and anticipated to be candidate EDCs feature one or more hydroxyl functions (bisphenols, benzophenones, alkylphenols, parabens…). Due to the presence of hydroxyl group(s) readily available for conjugation by phase II enzymes, such compounds are predominantly prone to undergo phase II (conjugative) rather than phase I (oxidative) metabolism, as previously demonstrated for bisphenols [8,9,10] and benzophenones [8,32]. The occurrence of phase I reactions was systematically checked in this study, but oxidative pathways were not found to be involved in the metabolism of BPS and BP2 in larvae or adult zebrafish, which is consistent with previous studies on the metabolic fate of bisphenol F [28] and of benzophenones in fish. These findings do not rule out the possibility that some phase I enzymes are functional in zebrafish larvae, as previously demonstrated for chemicals such as paracetamol and hydroxybupropion, among others [33].

For larvae, total body burden was lower for BPS (5.6 µg/g of BPS equivalents, wet weight) than for BP2 (22.9 µg/g of BP2 equivalents, wet weight). Accordingly, adjusted concentration factors (radioactivity in larvae/radioactivity in water) were also different (BPS: 22.0 ± 3.8; BP2: 93.3 ± 5.0). For BPS, this concentration factor (CF) of 22 was in the same order of magnitude as previously reported for BPA (CF: 27) and BPF (CF: 11) in a developmental study in which zebrafish larvae were exposed at 50 µM [28]. Qualitatively, after 72 h of exposure, water samples from larvae contained only the parent compounds. Conversely, radio-HPLC profiling of larvae extracts demonstrated an extensive metabolism of BP2 (87.3% ± 3.7% of extracts recovered as conjugates) and to a lower extent of BPS (34.2% ± 5.3%). This relied, for BP2, on higher glucuronidation and sulfation conversion rates, and on the occurrence of a minor double conjugate. None of these conjugates was found to be excreted back into water. Interestingly, a significantly higher rate of metabolic conversion of BP2, compared to BPS, was also previously observed in the zebrafish hepatic cell lines ZFL and ZELH-zfERs [8]. Likely enough, the higher Log*P* of BP2 (3.16, based on the American Chemical Society, 2007, calculated properties) compared to BPS (1.65 based on US EPA Estimation Program Interface (EPI) Suite; 1.9 based on XLogP3 from PubChem), could explain the easier uptake and bioavailability of BP2.

In adult zebrafish as well, marked qualitative differences were noticed between BPS and BP2 despite their chemical similarities. The ratio between the respective quantities of radioactivity in water and fish (apparent bioconcentration factor) was 1.7 ± 0.4 for BP2, and 0.6 ± 0.1 for BPS. For BP2, these results are consistent with the bio-concentration factor reported in a previous work in fathead minnows (*Pimephales promelas*) exposed to BP2 (0.4 µM) in a 15-day study (1.1 ± 1.0; [12]. Glucuronidation was the major pathway for BPS, with more than 78.5% ± 3.3% of the absorbed BPS converted into BPS-monoG, sulfation being a less preeminent pathway (7.7% ± 0.7%). Conversely, for BP2, sulfation (63% ± 1.6%) largely predominated over glucuronidation (22.3% ± 3.8%) in adults. The formation of a double conjugate (glucuronide + sulfate) was also characterized, but only for BP2. Unchanged (parent) compounds in adult extracts accounted for only 12.1% ± 4.3% (BPS) and 4.5% ± 0.9% (BP2), respectively. The comparison of the metabolic pattern of BP2 and BPS suggests the involvement of different sulfotransferases (SULT) and glucuronidases (UGT) in their respective biotransformation. Zebrafish UGTs 1A1, 1A7 and 1B1, and in particular UGT1A1 in the case of BPA, have been shown to be the major isoforms involved in the conjugation of phenolic and carboxylic compounds in HEK293T modified cells [34], and SULTs are known to participate in the metabolism of both endogenous and exogenous substrates. BPA sulfation in zebrafish has been shown to involve three SULTs from two families, namely SULT1 ST5, SULT 3 ST1 and SULT3 ST3 [35,36]. Although no direct analogy can be made with BPS, it was previously shown that the mRNA of SULT1 ST5 is weakly expressed from 0 to 72 hpf in zebrafish, whereas the mRNA of SULT3 ST1 is much better expressed at this developmental stage [37,38,39]. Temporal changes in genes expression encoding for XME could explain, at least partly, the differences in BP2 and BPS metabolic patterns observed between larvae and adults. For SULTs, at 72 hpf, the mRNA of SULT1 ST1, SULT2 ST2, ST3, and SULT3 ST1, ST2 are strongly expressed. Conversely, at 3 months of age, the mRNA of SULT1 ST3, ST4 and SULT3 ST1, ST2 are the most strongly expressed [37,38,39].

Remarkably, BP2 was more extensively metabolized into sulfo-conjugated metabolites than was BPS, particularly in adult zebrafish (Figure 3). The latter observation is consistent with the results already established with zebrafish cell lines (hepatocytes in primo-culture, ZFL and ZELH-zfERs cell lines) and with human cell lines (HepaRG, HepG2, MELN and T47D-KbLuc) [8] in a comparative BP2/BPS study. It is a strong hypothesis that sulfotransferases activities specifically expressed in gills explain the more extensive production of BP2 sulfo-conjugated metabolites in adult zebrafish, compared to BPS. For the latter molecule, no metabolites were detected in water. The overall proportion of BPS converted into metabolites was solely related to the metabolites found in the adult extracts (i.e., 0.35% ± 0.03% of the total radioactivity). The fate of BP2 was totally different, since all BP2 metabolites (except BP2 mono-glucuronides) were found in adult extracts as well as in water, in which they accounted for nearly 40% of the radioactivity. Consequently, despite structural similarities, BP2 was much more metabolized than BPS in adult zebrafish.

In addition to providing relevant and novel information regarding the metabolism of BP2 and BPS, our results have several implications regarding toxicity and estrogenic activity testing in the zebrafish embryo model. The study allowed to better characterize the life-stage dependent metabolic capacity of zebrafish. This aspect is critical since zebrafish embryos are now considered as an alternative model to adult experiments for toxicity testing [19,40] as well as estrogenic activity [6]. Differences in uptake, metabolism and toxicokinetics of xenobiotics between embryos and adults have often been hypothesized to explain the life-stage dependent toxicity or estrogenic activity of tested compounds, but have seldom been addressed experimentally [41,42]. While the overall uptake was low whatever the molecule and the life stage of development, marked differences in the concentration of residues in fish bodies were noticed with 40- and 50-fold higher concentrations of radioactivity per g of fish in larvae as compared to adults for BP2 and BPS, respectively. Based on these data, higher estrogenic activity of BP2 and BPS should be expected to be measured in zebrafish embryo assays, compared to adults. However, recent experiments have revealed that the effective concentrations of BP2 and BPS required for the up-regulation of ER-regulated genes are much higher in zebrafish embryos (brain aromatase B) than in adults (hepatic vitellogenin) [42,43]. It is likely that the toxicodynamics and toxicokinetics of the parent compounds may account for such differences. In this regards, development of physiologically based toxicokinetic (PBTK) models describing the uptake, metabolism and disposition of organic chemicals in zebrafish embryo and adult should help predict the effect of chemicals at various life-stages of development [44]. An important outcome of our study relies on the fact that it demonstrates the metabolic competence of the zebrafish embryo model, with extensive phase II metabolization capacities, and qualitatively similar sulfo- and glucurono-conjugated pathways for BPS as well as BP2, in embryos and adults. The ratio between sulfo- and glucurono-conjugation was clearly dependent upon the developmental life-stage (larvae vs. adult), reflecting the fact that phase II metabolic pathways could be differently activated in adult and larvae. Notwithstanding, in none of the zebrafish at adult or larval stage were phase I metabolites observed. This contrasts with sub-cellular models such as microsomes, which favor (in classical conditions of use) the formation of phase I metabolites, but are not necessarily representative of in vivo models.

## 4. Materials and Methods

### 4.1. Chemicals

Ring-labeled [3H]-benzophenone-2 (2,2′,4,4′-tetrahydroxybenzophenone; radiochemical purity: 99%, specific activity: 740 GBq/mmol) was purchased from American Radiolabeled Chemicals, Inc. (Saint-Louis, MO, USA). Ring-radiolabeled [3H]-bisphenol-S (4,4′-sulfonyldiphenol; radiochemical purity: 99.7%, specific activity 62.9 GBq/mmol) was supplied by Moravek Biochemicals, Inc. (Brea, CA, USA). Unlabeled benzophenone-2 (BP2, CAS #131-55-5, chemical purity: 97%) and bisphenol S (BPS, CAS #80-09-1, chemical purity: 98%) were purchased from Sigma–Aldrich (Saint Quentin Fallavier, France). Flo-Scint II and Ultima Gold liquid scintillation cocktails were purchased from PerkinElmer Life and Analytical Sciences (Courtaboeuf, France). HPLC-grade solvents (ethanol, methanol, dichloromethane, acetonitrile) were purchased from Scharlau (Barcelona, Spain). Ammonium acetate was purchased from Merck KGaA (Darmstadt, Germany). Ultrapure water produced with the Milli-Q system (Millipore, Saint Quentin En Yvelines, France) was used for preparing HPLC mobile phases.

### 4.2. Zebrafish Housing

Experiments were performed in accordance with the European Union regulations concerning the protection of experimental animals (Directive 86/609/EEC). Mature wild type zebrafish (*Danio rerio*, AB strain) were maintained in charcoal filtrated water at 28 ± 1 °C with a controlled photoperiod (14/10 h light/dark cycle). Water conductivity (350 ± 40 mS/cm) and water pH (7.5 ± 0.2) were checked. Fishes were fed SDS-400 (Special Diet Services, Dietex, Argenteuil, France) twice a day, seven days per week.

### 4.3. Biotransformation Studies in Zebrafish Larvae

After the spawning, fertilized zebrafish eggs were selected under a binocular magnifier. Each experimental group, consisting of 60 embryos, was exposed to BP2 or BPS dissolved in 50 mL water. Eggs were exposed with a controlled photoperiod (14/10 h light/dark cycle) in beakers placed in a water-bath to keep the temperature of exposure at 28 ± 2 °C. During the first 24 h, exposure consisted of either unlabeled BP2 or unlabeled BPS, at 1 µM. Water was renewed at 24 h, and from this time-point eggs were exposed for 72 additional hours, either to ^3^H-BP2 or to ^3^H-BPS, adjusted with unlabeled BP2 or BPS, respectively, to reach final concentrations of 1 µM and 8.41 × 10^5^ Bq per beaker. Chemicals were diluted in DMSO (Dimethylsulfoxide) before they were mixed with water, with a final concentration of 0.01% DMSO. The overall length of larvae exposure (96 h), was chosen to parallel the duration of exposure previously used with *cyp19a1b*-GFP transgenic larvae (EASZY assay), in a study addressing the estrogenic activity of EDC chemicals [6]. Control experiments were carried out in the same conditions with fish-farming water, without eggs, to simultaneously assess the degradation and/or microbial biotransformation of radiolabeled BP2 or BPS by micro-organisms, if any. All experiments were performed in triplicate.

After 96 h of exposure, water was collected and stored at −20 °C until radioactivity determination by direct counting using a Packard scintillation counter (model Tri-Carb 2200CA; Perkin-Elmer, scintillation cocktail: Ultima Gold) and radio-HPLC analysis. Larvae were euthanized in beakers using ice-cold water and then transferred into lysing Matrix D tubes containing ceramic spheres (MP Biomedicals, Santa Ana, CA, USA) and 500 µL acetonitrile:methanol:acetate ammonium buffer 40 mM pH 3.5 (6:3:1, *v*/*v*/*v*). They were crushed for 30 s using a high-speed homogenizer (FastPrep^®^-24^TM^, MP Biomedicals). After a centrifugation step (15 min, 4 °C, 1500× *g*), the supernatant was recovered. Two additional extractions were performed on the pellet using the same procedure. Supernatants were stored at −20 °C before radioactivity quantification and radio-HPLC analysis. Incubation beakers were rinsed with an ethanol/water solution, for which the radioactivity content was quantified using the scintillation counter, to complete the metabolic balance study.

### 4.4. Biotransformation Studies in Adult Zebrafish

#### 4.4.1. Exposure

The study of the fate of BP2 and of BPS in adult male zebrafish was carried out in fishes housed in 1.5 L tanks placed in an incubator, at 28 ± 2 °C, with a controlled photoperiod (14/10 h light/dark cycle). A biomass of 5 g of fish per liter of water was used, corresponding to 14–16 fishes/1.5 L. This biomass was selected based on preliminary experiments carried out to ensure an optimal balance between fish concentration and the use of a sufficient amount of radioactivity to perform further radio-HPLC analysis. With this biomass, preliminary studies demonstrated no changes in water parameters (dissolved oxygen, conductivity, pH and temperature), no mortality and no signs of suffering. During the first 4 days of exposure, fish were exposed to unlabeled BP2 or BPS, at 1 µM, with a complete renewal of water every day. During the last 3 days of exposure, fish were exposed either to ^3^H-BP2 or ^3^H-BPS adjusted with unlabeled BP2 or BPS to reach a final concentration of 1 µM and an amount of radioactivity of 1.009 × 10^6^ Bq per tank. Chemicals were diluted in DMSO before being mixed with water. The final concentration of DMSO in water was 0.005%. Control assays were carried out for each radio-labeled molecule in the absence of fish, but using fish-farming water, to assess the potential degradation and/or biotransformation of labeled parent compounds by micro-organisms. At the end of the exposure period, fish were pooled and euthanized in 100 mL ice-cold water, then were weighed and stored at −80 °C. Water was collected and stored at −20 °C. An ethanol/water solution (70:30, *v*/*v*) was used to rinse tanks and utensils. The quantification of radioactivity was carried out for tank water samples, water samples used to euthanize fish, as well as ethanol/water rinsing solutions. All experiments were performed in triplicate.

#### 4.4.2. Samples Preparation for Radio-HPLC Profiling

Tank water samples (2 mL each) were concentrated using a vacuum evaporator (speed Vac plus SC110A, Savant instruments, Fisher Scientific, Illkrich, France), with no heating, up to a volume of 100 µL, then were retaken in HPLC mobile phase A and were analyzed by radio-HPLC. Adult fish were extracted as follows: for each molecule and replicate separately, ca. 1 g of zebrafish (two fishes) previously stored at −80 °C was grinded in a ball grinder (ball mills MM400, Retsch^®^, Fisher Scientific) for 2.5 min at 28.5 Hz. The resulting powder was retaken with 8 mL/g CH_3_OH/H_2_O/CH_2_Cl_2_ (1:1:2 *v*/*v*/*v*), was shaken for 15 min, and centrifuged for 15 min (4 °C, 5000× *g*). The resulting aqueous and lipid phases were collected. This extraction was repeated twice. Radioactivity was quantified in both phases, and samples were stored at −20 °C until analysis.

### 4.5. Radio-HPLC Profiling

A Spectra P1000 HPLC pump (Thermo Separation Products, Les Ulis, France), coupled to an on-line radioactivity detector (radiometric flow scintillation analyzer Flo-One A500, PerkinElmer), were used to perform the radio-HPLC profiling of BPS and BP2 metabolites. Analyzed samples included water (larvae exposure experiments, adult exposure experiments, controls), as well as the aqueous phases of zebrafish larvae and adult zebrafish extracts. Organic phases and pellets from adult extracts were stored at −20 °C. The HPLC system, developed previously [8], consisted of a Zorbax SB-C18 column (250 × 4.8 mm, 5 µm, Agilent Technologies, Les Ulis, France) coupled to a C18 guard precolumn (18 × 4.5 mm, 5 µm, Macherey-Nagel, Hoerdt, France) maintained at 30 °C. Mobile phases A and B (1 mL/min) consisted of ammonium acetate buffer (20 mM, pH 3.5) and acetonitrile 95:5 *v*/*v* in A and 10:90 *v*/*v* in B. Two different 5-step gradients were developed for BP2 and BPS. These gradients have been described previously [8] and were used identically in the current study. Metabolites were quantified by integrating the area under the peaks monitored by radioactivity detection.

### 4.6. Enzymatic Hydrolyses

Aryl-sulfatase from *Aerobacter aerogenes* (type IV, Sigma-Aldrich) and bovine liver β-glucuronidase (type B1, Sigma-Aldrich) were used to confirm the formation of sulfo- and glucurono-conjugates, respectively. Bovine liver β-glucuronidase (150 IU in 300 µL 0.2 M sodium acetate buffer, pH 5) or arylsulfatase (0.15 IU in 300 µL of 0.01 M Tris buffer, pH 7.1) were added to 100 µL of each tested sample (water from larvae exposure, water from adult exposure, water from controls, supernatant from larva extractions, aqueous phase from adult zebrafish extractions). After 3 h at 37 °C under gentle shaking, hydrolysis assays were stopped by lowering the temperature of tubes in ice. Samples were mixed to 100 µL of mobile phase A prior to radio-HPLC analysis.

### 4.7. Radioactivity Quantification in Adult Zebrafish

Radioactivity in the different liquid samples was quantified by direct counting in a Packard scintillation counter (Model Tri-Carb 2200CA; PerkinElmer) using Ultima Gold as the scintillation cocktail. For all vials, sample quenching was compensated by the use of quench curves and external standardization. Radioactivity in whole fish was determined by complete combustion using a Packard oxidizer 306 (Perkin Elmer), prior to radioactivity quantification by liquid scintillation counting, as described above.

## 5. Conclusions

The comparative analysis of BP2 and BPS metabolic profiles in zebrafish models further supports the use of zebrafish embryo as a relevant alternative system in which toxicity and estrogenic activity can be assessed while still taking into account absorption and active metabolism of the tested substances. Overall, this study, together with our previous results on zebrafish cells [8], further argues for the relevance of zebrafish as a biological model for in vitro and in vivo integrative assessment of chemicals.
